# Barriers to and facilitators of partner notification for chlamydia trachomatis among health care professionals

**DOI:** 10.1186/s12913-014-0647-5

**Published:** 2014-12-20

**Authors:** Kevin ATM Theunissen, Pim Schipper, Christian JPA Hoebe, Rik Crutzen, Gerjo Kok, Nicole HTM Dukers-Muijrers

**Affiliations:** Department of Sexual Health, Infectious Diseases and Environmental Health, South Limburg Public Health Services, PO Box 2022, 6160 HA Geleen, The Netherlands; Department of Medical Microbiology Maastricht Infection Centre (MINC), School for Public Health and Primary Care (CAPHRI), Maastricht University Medical Centre (MUMC+), PO Box 5800, 6202 AZ Maastricht, The Netherlands; Department of Health Promotion, School for Public Health and Primary Care (CAPHRI), Maastricht University Medical Centre (MUMC+), PO Box 5800, 6202 AZ Maastricht, The Netherlands; Department of Work & Social Psychology, Maastricht University, P.O. Box 616, 6200 MD Maastricht, The Netherlands

**Keywords:** Partner notification, Chlamydia trachomatis, Barriers, Facilitators, Public health

## Abstract

**Background:**

Partner notification (PN) is an essential case-finding tool in the management of sexually transmitted infections (STIs). Yet, data on the effectiveness and factors impacting implementation of PN in the Netherlands are lacking. With the aim of further exploring and improving the PN process, the current study assessed perceived barriers and facilitators among health care professionals in the STI clinical setting. In particular, we explored the management of PN in young heterosexual patients diagnosed with Chlamydia trachomatis (Ct).

**Methods:**

We conducted semi-structured interviews among 22 health care professionals (response rate 52%) from 5 of the 8 national STI clinics in the Netherlands. We carried out qualitative content analysis using a framework approach. All participants were nurses, aged mid 20’s to late 50’s, and all but one were female.

**Results:**

All health care professionals felt comfortable discussing PN. Other perceived facilitators for PN included: time, one-on-one consultations, interviewing skills (i.e. Motivational Interviewing) and a proactive helping style. Important barriers were identified as: sub-optimal guidelines, inaccurate sexual history, a lack of feedback regarding the motivational strategies that were used, and the lack of feedback regarding overall PN effectiveness. The health care professionals placed an emphasis on the care and treatment of the individual index patient rather than on discussion of PN, or on motivating and helping patients to engage in PN.

**Conclusions:**

Health care professionals identified several barriers that need to be overcome, and facilitators which need to be maintained. Future efforts should concentrate on introducing PN protocols, providing feedback on both the effectiveness of strategies used by health care professionals, and on the PN process as a whole, and educating health care professionals about Motivational Interviewing strategies. Moreover, the possible implementation of an Internet-based PN system should be explored.

## Background

Partner notification (PN) has an essential role to play in the management of sexually transmitted infections (STIs), both for the individual (i.e. in terms of the prevention of re-infection and complications) and the community (i.e. in terms of transmission interruption) [1,2]. The PN process entails four steps: 1) a health care professional discusses PN with an STI-positive patient and explains the possible infection risk for sex partners, 2) the sex partner is then identified, 3) notified, and is finally 4) tested, treated and educated [3]. The primary strategies used to notify partners include provider referral and patient referral. Provider referral involves the provider contacting the patient’s partner(s). Compared to patient referral -- where the patient notifies his/her sex partners -- provider referral has been shown to be more effective at increasing the number of sex partners who are subsequently tested and treated [3-5].

When all 4 PN steps are carried out successfully, PN is an extremely effective tool in STI prevention, as it enables a high-risk population to be targeted, tested and treated. However, barriers at the health care professional, patient and organisational levels can disrupt the process at every step [1]. Data about PN barriers among public health care professionals (i.e. nurses) are scarce, and most research is conducted among General Practitioners (GPs). Previous research investigating PN barriers as perceived by GPs identified several important barriers at step 1 (i.e., discussing PN with the patient), including: time pressure, lack of financial reimbursement, and provider discomfort [6,7]. While GPs are generally supportive of PN, they can be unaware of, or misunderstand, their own role in PN; for example, they may assume that PN will be performed by local health care services [6,8,9]. Most GPs prefer patient referral [5,6], as provider referral is perceived as being both costly and time consuming [2,5].

In the Netherlands, public health STI clinics are responsible for approximately 30% of STI care and the large majority of PN [10]. The organisational structure and scope differs between medical (i.e., among GPs and medical specialists) and public health care (i.e., STI clinics) settings. The latter are often described as being more concerned with populations than with individuals, and with prevention and care more than with cure [11,12]. There may well be different barriers to and facilitators of PN in medicine as compared to public health care, yet data on public health care professionals who perform PN in STI clinics are scarce, and any available data focuses on STI/HIV in general [13].

This study examined the barriers to and facilitators of PN, as identified by public health care professionals, in relation to young heterosexual patients diagnosed with *Chlamydia trachomatis* (Ct) who had visited an STI clinic for treatment. In the Netherlands, PN is not mandatory or enforceable by health care professionals. The role of the health care professional is to motivate and help Ct positive patients to identify and notify their sex partners. PN can be initiated when a STI test is performed, when a patient is informed about a positive test result by telephone, or when a patient visits the STI clinic for treatment. In cases where a patient agrees that the health care professional may notify his or her sex partner(s), the health care professional will telephone or send a text message. Other tasks performed by STI clinic health care professionals include sexual health consultations, STI testing, treatment, and education. Their professional role description (as part of a public health service) includes the protection of the community as a whole (i.e. sex partners). In the current study, we focused on Ct because it is the most common STI in patients younger than 25 years old, with an estimated prevalence of 17% in 2013 in the Netherlands [14]. Young patients have consistently high rates of risky sexual behaviour and, in terms of reproductive morbidity, potentially bear the largest burden of STI sequelae [15]. It is our intention that findings from the present study will inform a more effective PN process i.e., improve the prevention of re-infection and complications and interrupt transmission.

## Methods

### Design and setting

In order to study the perceptions of health care professionals, a qualitative method (i.e. semi-structured interviews) was applied. Therefore, this article adheres to the RATS guidelines on qualitative research [16]. The study took place among health care professionals (i.e., trained STI clinic nurses) as they performed PN in public STI clinics. Participating health care professionals provided written informed consent, and the Medical Ethics Committee of Maastricht University reviewed and approved this study (reference number 13-4-054).

### Recruitment

Between March and June 2012, an invitation letter with a short explanation of the study was sent to the email addresses of 42 nurses who had performed PN at their STI clinic for a period of at least six months. Email addresses were obtained from contact information that was available to the researcher, with contacts covering all 8 Dutch coordinating STI clinics. Within two weeks, 22 trained public sexual health care professionals (response rate of 52%) from 5 of the 8 national STI clinics in the Netherlands had been recruited. As thematic saturation (i.e., the point at which no new themes emerge) was reached within this sample, no email reminder was send to the nurses who did not respond to the first invitation email.

### Data collection

Interviews were audio-recorded with the permission of the health care professionals, and conducted face-to-face (n = 11) or over the telephone (n = 11) by the interviewers. Both interviewers were affiliated to the Public Health Service South Limburg and the University of Maastricht, and had been trained in conducting semi-structured interviews and qualitative analyses. PS is a medical student and KT is an experienced researcher. The telephone and face-to-face interviews lasted, on average, 22 minutes. Recordings were assigned a number to ensure confidentiality. Numbers and corresponding names were kept in a locked file. Data were collected using a semi-structured interview protocol consisting of 17 questions. This protocol was constructed in line with expert opinion, a comprehensive review of the literature, and (inter)national guidelines. At the beginning of each interview, the interviewers stated that all questions were related to Chlamydia diagnosis in young heterosexual people. Examples of questions are: At what stage in the process is PN carried out, and how do you perform PN?; Which strategies do you apply during PN?; How do you feel when performing PN?; Which barriers do you experience during PN?; and Which facilitating factors do you experience during PN? The interview protocol was piloted among health care professionals before implementation. Saturation (i.e. the point at which no new themes emerged in the interviews) occurred after approximately 17 interviews, and later interviews served to confirm themes identified earlier in the analysis.

### Analyses

The audio-recorded interviews were transcribed verbatim in Dutch. Transcripts were analysed independently by PS and KT using the ‘framework’ approach [17], which involves structured stages of data management, descriptive accounts and explanatory accounts. Furthermore, several transcripts were explored in detail, in order for PS and KT to become familiar with the data, after which open coding was applied. Codes were then grouped into categories in an iterative process, until no additional codes emerged. Eventually, categories and codes were applied to subsequent transcripts. A spreadsheet was used, which also included illustrative quotes, to find associations within categories and explanations for these associations were sought. Eventually, six categories were made: health care professional, patient, and organisational barriers and facilitators, respectively. Any disagreements found in the analysis were resolved through discussion, and consensus was reached by PS and KT consulting a third party (i.e. ND). Quotes that were used to illustrate the findings were translated into English and checked by the interviewers (PS and KT) to ensure accuracy.

## Results

### Sample characteristics

Twenty-one of the 22 participants were female, representing the sex ratio of staff in national STI clinics. The participants were aged between 24 and 55 years old. All had at least 6 months of experience, and all had received training in Motivational Interviewing (MI), which comprised part of their job education program.

**Figure 1:**
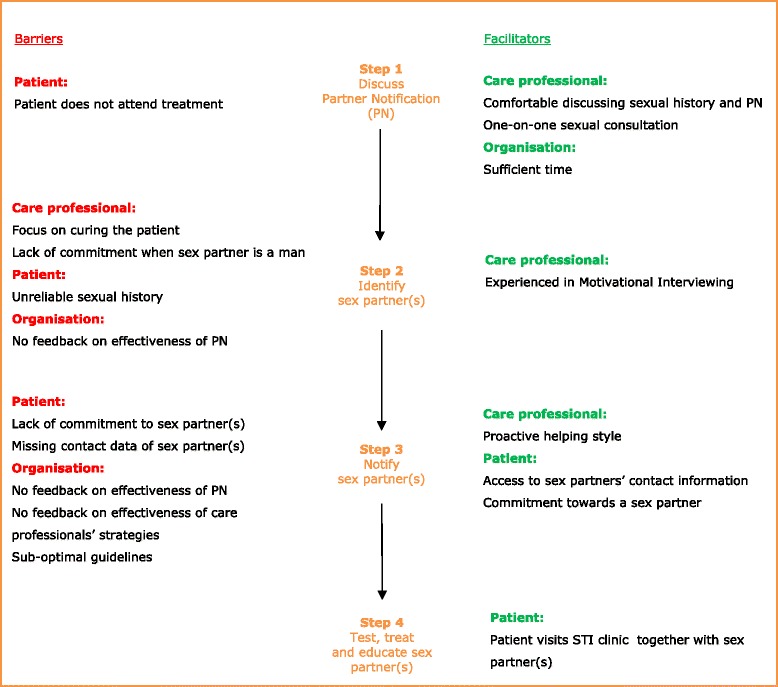
**Care professionals’ perceived barriers to and facilitators of partner notification derived from semi-structured interviews (n = 22).**

The barriers to and facilitators of PN (as perceived by health care professionals) are outlined below, using quotes to illustrate our findings. An overview of all barriers and facilitators is provided in Figure 1.

### Health care professional barriers

Health care professionals primarily mentioned barriers in steps 2 and 3 of PN, i.e., the process of identifying and notifying sex partners. Some participants indicated awareness of the PN-related goal of public health care, that is, the prevention of STIs in the community. However, nearly all health care professionals expressed a bigger commitment to curing the (index) patient rather than interviewing the patient about PN and helping him/her to notify (by themselves or with a staff member) sex partners of their exposure to an STI.

*“My feeling is that it [notification of partners] is really the patient’s own responsibility, although I do work at a Public Health Care Service, whose job it is to identity and treat as many people as possible”.*

[Female no. 13, mid 30’s, Region 5]

*“For me, the patient and his questions come first, because he is sitting in front of me, he is top of the list”.*

[Female no. 16, late 50’s, Region 1]

Health care professionals felt that placing too much pressure on PN could drive current patients away from future testing, in which case access to a high risk population would be lost.

*“The community is important, but you should not scare the patients away from public health care”.*

[Female no. 14, age unknown, Region 5]

Moreover, client referral, as opposed to provider referral, was preferred by almost all health care professionals. They viewed the notification of sex partners as the responsibility of the patient.

*“My task is to convince and to inform, the actual notification is the client’s responsibility”.*

[Female no. 22, mid 40’s, Region 3]

Some of the health care professionals were more committed to protecting female partners than male partners of patients, as females have a higher disease burden.

*“… it [PN] is important, because girls could become infertile. To be honest, because of this, I take a more active attitude with girls, as they may suffer more serious consequences than men”.*

[Female no.5, late 20’s, Region 1]

### Health care professional facilitators

All of the health care professionals felt comfortable assessing sexual history and discussing PN (i.e. PN step 1). Generally, PN was practised one-on-one, and the process was not disrupted by the presence of third parties.

*“I think this [discussing PN] is a task that belongs to us. I do not have any problems with discussing it [PN].*

[Female no. 13, mid 30’s, Region 5]

*“I try to see all my patients one-on-one without the presence of a partner… so you know they will provide you with complete information… it will be easier to ask more in-depth questions about their sex partners from the past 6 months”.*

[Female no. 6, mid 20’s, Region 1]

Furthermore, all health care professionals had been trained in Motivational Interviewing, which they felt to be an advantage at moments in which a patient’s willingness to identify sex partners was low. When a patient appeared reluctant to notify their partners themselves, for instance because they were afraid to do so , health care professionals contacted his/her partner(s). However, this happened only occasionally, because patients often indicated that they preferred to notify their sex partner(s) themselves.

*Interviewer: “How often do you apply Motivational Interviewing techniques during a conversation?”*

*Health care professional: “Sometimes, because you do not need to apply it every time…when I have many doubts about someone’s willingness to comply. In such moments it will help you to determine what someone thinks of it [Partner Notification], and it will indicate how far you can go with this person”.*

[Female no. 22, mid 40’s, Region 3]

The scope of public health care is to protect the community as a whole (i.e. sex partners) and not merely focus on the treatment of individuals. In line with this scope, it is notable that one health care professional mentioned that her professional role was to convince people to notify their partners and to use a more proactive helping style towards patients. As a result, she was often asked by patients to notify sex partners on their behalf.

*Health care professional; “When I started working here, I felt it [PN] was the client’s responsibility. Meanwhile I think it is entirely our [health care professional and public health care] job to convince people to notify their partners”.*

*Interviewer: “Is it only about informing partners, or is it more?”*

*Health care professional:” Of course informing -- that was also the case when I started here. But I mean more. I mean estimating whether you should take control in the interview and use a more directive style”*

[Female no. 10, early 50s, Region 1]

*Interviewer: “How many people request that you notify their partners, and how many people do it themselves?*

*Health care professional: “I try my best to achieve 50 per cent, but so far this has proven impossible. No, I think 40 per cent request me to take over a big part. I convince them in an early phase, the best way in current practice”.*

[Female no. 10, early 50s, Region 1]

### Perceived patient-related barriers

Some health care professionals mentioned that PN can be hindered when opportunities to discuss PN (PN step 1) are inadequate, especially when face-to-face contact is not possible.

*“… When a patient has tested positive for Chlamydia and refuses to return for treatment. That is, those patients who consider returning for treatment a nuisance…They just want a prescription, that’s it. It really annoys me, because I will have to discuss PN over the phone and make it quick at that”.*

[Female no. 3, mid 20’s, Region 1]

Even though health care professionals felt comfortable discussing PN, they were sometimes confronted with a lack of commitment among patients towards sex partners when discussing a Ct diagnosis. Reasons for this lack of commitment mentioned by the health care professionals included feelings of anger, fear and embarrassment among patients towards their sex partners.

*“It is difficult when a client in front of you does not feel any commitment towards the person they have to notify -- for example, because their partner was unfaithful and got infected with Chlamydia. This is an obstacle we face during Partner Notification”.*

[Female no. 19, late 30’s, Region 1]

In addition, health care professionals took into account that sexual history may be unreliable due to patients providing socially desirable answers. Health care professionals indicated that in such cases it is challenging to find opportunities to discuss PN optimally.

*“Basically, a sexual history is unreliable. People pimp up their stories to appear more flattering”. So, I always assume that they have more contacts than they reveal.*

[Female no. 4, early 50’s, Region 1]

*“It [dealing with socially desirable answers] is sometimes even harder than trying to persuade someone who frankly says ’I won’t notify’, because with the latter I can at least start the conversation”.*

[Female no. 3, mid 20’s, Region 1]

An additional barrier to PN included patients not having their partners’ contact information.

*“Sometimes, it [PN] is impossible, because people had a one night stand or visited a disco and do not have a telephone number; in such instances, there is simply no room for PN”.*

[Female, no 22, mid 40’s, Region 3]

### Perceived patient-related facilitators

Some health care professionals mentioned that, at the time of treatment, index patients often claim to have already notified their partners. Health care professionals stated this is often the case when patients have high feelings of responsibility toward their sex partners (i.e., are in steady relationships).

*“When they [Chlamydia positive patients] visit us for treatment, they quite often indicate that they have already notified their partners.*

[Female no. 15, age unknown, Region 3]

Some health care professionals said that patients occasionally visit the STI clinic with a sex partner during a treatment consultation. In current practice, both the patient and the sex partner will receive treatment (i.e., PN step 4).

*“When someone brings along a partner to a treatment consultation, both of them will be treated”.*

[Female no. 15, age unknown, Region 3]

### Perceived organisational barriers

Health care professionals are not obliged to register the PN process in the Electronic Patient Record, and therefore no feedback on the effectiveness of PN outcomes and PN techniques used was available. The majority of the health care professionals did not know whether their PN techniques were effective, and some felt that their own contribution in PN was limited. In addition to this, health care professionals were unaware of whether patients’ sex partners were subsequently tested, treated, and educated (PN step 4).

*“Often young people want to notify their partners themselves, and of course we do not have any idea whether they actually do so, or if they actually manage to get in touch. This makes the process difficult. Actually, you would like to count who [sex partners] is visiting you and if they are tested”.*

[Female no. 21, mid 30’s, Region 3]

*“To be honest, I do not know, never measured this…. I have never received feedback on it [Partner notification]”.*

[Male no. 12, late 40s, Region 4]

*“Whether it [PN] works or not, we do not know. We do not monitor whether a sex partner has been notified or has actually been tested.*

[Female No.1, mid 20’s, Region 1]

*“There are many factors that play a role; my contribution [in the notification of partners] is only a small one”.*

[Female no.4, early 50s, Region 1]

Health care professionals also stated that current guidelines do not specify in detail which motivational strategies to use, which PN procedure to follow, or which referral strategy is preferred; the guidelines only include a recommended recall period for tracing back sex partners. However, the health care professionals also mentioned variation in the use of these recall periods, suggesting that some staff members did not adhere to the guidelines.

*“The recall period of sex partners is stated on paper, but the way you should do this [PN] is not. Everyone applies Motivational Interviewing their own way”.*

[Female no. 22, mid 40’s, Region 3]

*“We do not strictly follow the national guidelines as stated during PN. We ask young people if they know who infected them… If they don’t know, we ask them to recall sex partners from the past 6 months”.*

[Female no. 17, early 60’s, Region 2]

### Perceived organisational facilitators

Almost all health care professionals reported that their STI clinics provide them with sufficient time to conduct and discuss PN (PN step 1).

*“There is sufficient time! Sometimes you need 5 minutes and on other occasions you need half an hour”.*

[Female no. 18, early 50’s, Region 1]

## Discussion

The semi-structured interviews we carried out among health care professionals in national public health STI clinics in The Netherlands revealed several barriers at the health care professional, patient, and organisational levels. These barriers may hinder the PN process (in which PN is discussed with the patient and sex partner(s) are subsequently identified, notified, tested, treated and educated). Important barriers were identified as: no-shows at the treatment stage (which was the most important moment to discuss PN), a focus on curing patients, less of a perceived need to conduct PN in male sex partners, a perceived lack of commitment among patients towards sex partners, missing contact data of partner(s), unreliable sexual history, a lack of feedback on the effectiveness of the PN process and on the strategies used by health care professionals, and sub-optimal guidelines. In addition to these barriers, important facilitators of PN were identified as: feelings of being comfortable discussing sexual history and PN, one-on-one consultation, sufficient consultation time, a proactive helping style, being experienced in Motivational Interviewing, patient commitment towards sex partner(s), and having sex partners attend the clinic together with the patients

While barriers among GPs concentrated on the discussion of PN (i.e. step 1 in the PN process), barriers among STI clinic professionals were mostly related to steps 2 and 3 (see Figure 1). Public health care is complementary to medicine, and has a different scope and organisational structure. Public health care aims to protect the community; it benefits individuals by providing treatment and preventing re-infection. Public STI clinics are non-profit organisations that provide free care and employ experienced health care professionals (i.e., staff who are experienced in Motivational Interviewing and sexual consultation). Contrary to the public health scope, most of the health care professionals in this study were more committed to curing patients than to preventing STIs in sex partners. Potentially, such a curative approach is maintained as the health care professionals do not have any information on the effectiveness of PN on the community, or any feedback on the PN process and the strategies they have implemented. As a possible result, health care professionals may feel less responsible for PN and the process of contacting and notifying sex partners on behalf of the patient. Currently, almost all notifications at Dutch STI clinics appear to be carried out by patients (i.e. patient referral) and not by health care professionals, as revealed during the interviews. Patient referral has generally been observed as the most common PN practice in patients with STIs [5]. However, its effectiveness is not known [18], due to the frequent absence of recorded PN outcome data (i.e. data regarding whether partners are notified, tested and treated) [2,18,19]. It is expected that patients will often fail to notify sex partners, because of the stigma surrounding STIs/HIV, and associated feelings of embarrassment or fear [20]. Therefore, barriers and facilitators surrounding PN, as identified by Ct positive patients and their partners, should also be considered when improving PN implementation in practice. While Motivational Interviewing was mentioned as a facilitator among health care professionals, there are differences in how well such techniques are applied; differences may be related to age, experience and/or personal attitude. Almost all health care professionals in our study merely informed the client about PN, while only one health care professional discussed PN and used a more proactive helping style in order to examine and resolve problems during PN. Notably, this health care professional was asked by almost half of the patients to notify sex partner(s) on their behalf (i.e. provider referral). Provider referral has been found to be more effective [4,18] and is thus important in the management of re-infections and the screening and testing of sex partners. Professionals can use email, text messages, telephone and outreach approaches such as face-to-face conversations to inform sex partners. However, provider referral is labour intensive, and a combination of different PN methods has therefore been recommended in the literature [18].

A low level of commitment towards sex partners (on the part of the patient) has previously been identified as a barrier in the PN process [21]. Health care professionals in our study identified low commitment as a barrier to the notification of patients’ sex partners. However, a previous study has demonstrated that young female and male patients who blamed their sexual partners for acquiring an STI infection still felt morally obliged to notify them [20]. The notification of sex partners may be hampered by patients under-reporting the number of sex partners in an attempt to provide socially acceptable answers – or simply forgetting [22].

The results of this study underline the fact that national and international guidelines about PN contain only general recommendations [23,24]. Guidelines do not specify which motivational strategies to use, which PN procedures to follow, or which referral strategy is preferred and how exactly to implement it. This lack of specific instructions was also reported to be a barrier among GPs [7]. Sub-optimal guidelines may lead to misconceptions about best practice, job roles, and responsibilities [7].

A different approach towards partner management, called Expedited Partner Therapy (EPT), has recently gained attention in the literature [25]. In this approach, partners are treated without a personal assessment. Although EPT decreases the number of PN steps necessary, and could therefore potentially optimize the PN process, some barriers identified in this study could also hamper the implementation of EPT. Examples of such barriers include a focus on curing the patient and the lack of commitment among patients towards sex partner(s). Future studies are needed to map the barriers and facilitating factors in both providers and the public regarding EPT.

Since the results of this study became clear, discussions have taken place among health care professionals about their emphasis on patient care rather than public health, and also about the absence of outcome measures to determine effectiveness. Nationally, there is an ongoing public debate about these issues, and the professional community has been informed. Currently, national PN protocols are being re-written and regional PN reporting systems have been developed, taking into account the findings presented in this study.

### Recommendations

It is important to use, improve, and maintain current facilitators of PN. On the other hand, perceived barriers to PN should be tackled (see Table 1). From an organisational perspective, future efforts need to concentrate on addressing the public health care goals of public sexual health care professionals, focusing especially on their responsibility towards the community. For example, this could involve the development of PN protocols that encourage the notification of sexual, and possibly also social, networks. Furthermore, attention should focus on Motivational Interviewing, which has been shown to improve skills and behaviour of health care professionals in dealing with patients’ resistance towards PN [26]. PN training using MI as a useful tool should therefore be included in the education of health care professionals. In addition to this, recall of sex partners is likely to increase when professionals are better trained to motivate patients to contact sex partners, or when care professionals are more proactive in helping patients in the notification process (i.e., provider referral)[4,5,18,22,26]. Since no feedback on the effectiveness of PN outcomes and PN techniques used is available, future efforts should also include developing ways to provide feedback to staff, which in turn could have a positive effect on their feelings of responsibility, and address their feelings of being ineffective. The frequent absence of recorded PN outcome data could be tackled by implementing a centralized and standardized collection of PN data. Regional and/or national PN reporting systems shared among all stakeholders who perform PN should be developed to determine, for instance, rates of notification, test, positivity and treatment among partners. One option, which was mentioned by some of the health care professionals, and has also been identified in the literature [27], would be to implement an internet-based PN system (i.e., e-mail and text messages) which can be used by both clients and health care professionals; this strategy would take advantage of a communication technology that is increasingly utilised [28]. An initiative of the Public Health Care Service South Limburg is to test and implement an internet-based system called SafeFriend [29]. Young people at risk for Ct will be motivated via their sexual and social networks (i.e. e-mail and text messages) to get tested for Ct, and offered home-based test kits. As shown in a systematic review [5], home-based test kits improve the effectiveness of PN by increasing the number of partners tested.

**Table 1:** **Recommendations to improve partner notification**

**Topic**	**Recommendations**
PN Guidelines	PN guidelines should provide concrete steps for health care professionals in terms of how they can address public health goals
PN training	Motivational Interviewing should be specifically addressed in training for PN
Discussing PN	Organisations should encourage and facilitate inter-professional discussions on best practice regarding PN
Feedback on PN	Information systems should be created and implemented that provide feedback on PN outcomes to health care professionals and policy makers

### Limitations

Some limitations need to be considered when interpreting the data. First due to logistical reasons, we decided to conduct the interviews in participating clinics outside South Limburg via telephone rather than face-to-face (interviews were conducted face-to-face in participating South Limburg clinics). To minimize the difference between the verbal and non-verbal communication of the interviewers, a protocol was used for each of the interviews. Data showed that there were no notable differences in the themes raised by the interviewees across the face-to-face or telephone interviews. Second, to minimize the possibility of receiving only socially desirable answers, the interviewers emphasized the confidentiality of data before and during the interview. Third, barriers and facilitators as perceived by health care professionals were studied only in relation to PN in young heterosexual people infected with Ct (i.e., the largest group of STI clinic patients in the Netherlands). Therefore, it is unknown whether results can be extrapolated to other target groups (i.e. men having sex with men, or commercial sex workers) and other STIs (e.g. Syphilis or HIV). Fourth, the experience that a health worker has (i.e. number of years working in a clinic) may play a role in the PN process. Positive and/or negative experiences over time could influence the attitudes and self-efficacy of health care professionals towards PN. Although we did not have exact data relating to employment years, all participants in the present study had at least six months PN experience in an STI clinic setting. Finally, the results of this study were not presented to the participants for confirmation. Nevertheless, at the end of each interview, participants were given the opportunity to ask questions and/or give comments concerning the interview.

## Conclusion

The results of this study carried out among Dutch STI clinics provides insight into the challenges and facilitators at the health care professional, patient, and organisational levels. These applied mainly to steps 2 and 3 of PN, i.e., the identification and notification of sex partner(s). In order to overcome these barriers and maintain facilitators --and thereby optimize PN -- efforts should be made to focus more on the public health care goals of STI clinical practice, especially on the aim of protecting the community. Examples of ways in which these goals can be reached include: introducing PN protocols, providing feedback on the effectiveness of strategies used by health care professionals, and on the PN process as a whole, education in the use of Motivational Interviewing strategies, and the possible implementation of an Internet-based PN system.

### Ethical approval

The Medical Ethics Committee of Maastricht University reviewed and approved this study (reference number 13-4-054).
